# Heteroblastic Inflorescence of *Lamium amplexicaule* L. in Egyptian Flora

**DOI:** 10.3390/plants12051028

**Published:** 2023-02-24

**Authors:** Wafaa M. Amer, Najla A. Al Shaye, Mahmoud O. Hassan, Maha H. Khalaf

**Affiliations:** 1 Department of Botany and Microbiology, Faculty of Science, Cairo University, Giza 12613, Egypt; 2 Department of Biology, College of Science, Princess Nourah bint Abdulrahman University, P.O. Box 84428, Riyadh 11671, Saudi Arabia; 3 Department of Botany and Microbiology, Faculty of Science, Beni-Suef University, Beni-Suef 62521, Egypt

**Keywords:** *Lamium amplexicaule*, heteroblastic inflorescence, cleistogamy, chasmogamy, genetic diversity, phenoplasticity

## Abstract

*Lamium amplexicaule* L. (Family: Lamiaceae) is a cosmopolitan weed whose eradication is challenging. The phenoplasticity of this species is related to its heteroblastic inflorescence, which has not received adequate research worldwide in its morphological and genetic aspects. This inflorescence hosts two flower types, a cleistogamous (CL: closed flower) and a chasmogamous (CH: opened flower). This species subjected to detailed investigation is a model species to clarify: (1) the existence of the CL and CH flowers in relation to the time and individual plants. (2) the predominant flower morphs in Egypt. (3) the morphological and genetic variability between these morphs. Among the novel data retrieved from this work is the Presence of this species in three distinct morphs coexisting during winter. These morphs showed remarkable phenoplasticity, particularly in flower organs. Significant differences were observed between the three morphs in pollen fertility, nutlets productivity and sculpture, flowering time, and seed viability. These differences were extended to the genetic profile of these three morphs assessed by the inter simple sequence repeats (ISSRs) and start codon targeted (SCoT). This work highlights the urgent need to study the heteroblastic inflorescence of crop weeds to facilitate its eradication.

## 1. Introduction

*Lamium* L. is one of 236 genera of the family Lamiaceae (subfamily Lamioideae), and it includes approximately 38 annual and perennial species according to its circumscription. Its phytogeographic region extends from Northern Africa to Eurasia. Its diversity center is situated in the Irano-Turanian and Mediterranean areas, evidenced by the fact that there are 47 *Lamium* taxa endemic to Turkey [1]. 

In Egypt, *Lamium* L. is a monospecific genus represented by *L. amplexicaule* L. [2,3], and subgenus *Lamium* L. section *amplexicaule* [4]. This species was recorded in Egyptian flora since [5] and verified by [2,3]. It is distributed as a common weed in winter crops and on the canal banks of the Nile River, the Mediterranean coastal stripe, and Sinai [2,3].

However, *L. amplexicaule* is native to the Egyptian flora [3]. It was naturalized in the USA as a prevalent weed in ≥50 summer and winter crops. It becomes problematic for the croplands [6]. The species’ phenoplasticity may increase its eradication efforts [7]. Also, this species has been reported as invasive in different countries and caused significant reductions in wheat productivity, inducing problems to the ecosystem and cropland in Iran [4]. Sheep and horses grazing on this species have also experienced mild-neurological issues [8], and cattle in Australia have been poisoned by it [9]. Accordingly, comprehensive data about this naturalized and invasive species is of hot request. 

*L. amplexicaule* shows extensive phenoplasticity, including the presence of heteroblastic inflorescence, which presents the coexistence of two flower types (representing the two mating systems) in the same individual, namely, the cleistogamous flower (CL; closed), in which the pollination is “Autogamous” and the chasmogamous flower (CH; opened) with cross-pollination (Allogamy), which is either “Geitonogamous” (pollination between flowers in the same individual) or “Xenogamous” (pollination between flowers of different individuals). CL and CH flowers have been observed in the same individual in California [10] and Central Japan [11]. Further, in Louisiana, [6] reported the Presence of three types of flowers in *L. amplexicaule* inflorescences, namely CL, CH, and pseudo-cleistogamous flowers (CL + CH; both opened and closed). In a single plant, the production of the mixed mating systems (CL and CH flowers) is referred to as dimorphic cleistogamy [12]; cleistogamy was earlier termed by [13] as true cleistogamy. 

*Lamium amplexicaule* grows in different seasons based on its geographical distribution. It is a summer weed in western Canada, while in the Southern USA, it is predominantly a winter weed, and its seeds can germinate in both autumn (August–October) and spring (March–May) [9]. However, heteroblastic inflorescence in *L. amplexicaule* shows inconsistent CL and CH flowers/individuals’ percentages. For instance, in France, [14] reported that the proportions of CH flowers varied in response to environmental conditions. A higher CH proportion was noticed when the plants were exposed to warm temperatures and long photoperiods in spring and a lower ratio in autumn when these conditions were opposite. And in N & S France [7], the dominance of CH/CL flowers was reported in the early season and shifted later to CL flowers when pollinators were limited.

The genetic diversity within and among populations with different mating systems (CL and CH flowers) is not yet fully understood. Inter simple sequence repeats (ISSRs) are one of the effective molecular markers applied to study genetic diversity within and among populations [15,16,17]. ISSRs were used to clarify the genetic diversity within and among populations of *Brassica tournefortii* morphotypes [18]. Furthermore, [4] used ISSR markers to study the genetic diversity of *L. amplexicaule* populations from Iran; the results revealed that the diversity within and among populations was 67% and 33%, respectively. Start codon targeted (SCoT) was recently utilized as an additional marker to ISSRs. SCoT was developed based on the short-conserved DNA region flanking the ATG start codon (or initiation) that is conserved in all genes [19]. SCoT has many advantages, such as low-cost, simple applicability, high polymorphism, generating extensive genetic information [20,21], good reproducibility [22], and application without previous genomic information. SCoT also has the advantage of being able to assess the genetic diversity among the small, diverse sets of rice genotypes [19]. Applications of SCoT could provide further information to help understand the genetic diversity of the investigated *L. amplexicaule* populations.

To the best of our knowledge, no previous studies have been conducted to clarify the species’ heteroblasticity and the genetic profile/s of the different morphs of the weed species with heteroblastic inflorescence to facilitate its eradication. This study aimed to investigate *L. amplexicaule* populations as a model species to clarify the following points: (1) Are the CL and CH flowers present in the same or separate individuals, and is this coexistence at the same time or at different times? (2) What is Egypt’s predominant flower morph (if any)? (3) What are the phenological and genetic profiles of each morph? (4) Is there any difference in seed productivity and pollen fertility between the different flower types (CL and CH)? and (5) What is the offspring flower morph relationship to their parental seeds? 

## 2. Results

### 2.1. Species Morphology

Plant morphology: annual green herb; sparse-densely pubescent 30–63 cm height. Stem quadrangular, ascending or decumbent, bearing basi-branched, green to pinkish branches. Leaves; (2.5-) 7–30 × (2.6-) 7–25 mm, ovate reniform to suborbicular, usually crenate to palmately lobed with obtuse-rounded lobes, petioles (0.5-) 1–1.5 (−3) cm. Floral bracts; (5-) 7–25 (−30) × (6-) 10–37 mm, *amplexicaule* with conspicuous trichomes and palmate venation, cordate-orbicular to reniform, margin irregularly crenate or upper lobes sometimes profoundly incised. Inflorescence verticillate, in terminal and axillary positions, 4–10 (−25) flowers (traced as cleistogamous (CL; closed flower) and chasmogamous (CH, opened), the distal flowers rudimentary. Flowers: bisexual, tubular, and bilabiate. Calyx: green and persistent, tubular-campanulate, (4-) 6–7 (−8.5) mm long, with 5-triangular teeth (1.5–4.5 mm/each), with non-glandular trichomes. Corolla: (12-) 15–20 (−28) mm long, (1-) 3–5 times as long as the calyx, purplish-pink but rarely white, purple spots on the lower lip traced in one morph. Upper lip: two united petals forming hood-like, with trichome patch outside, (2-) 4–6 × 3–4 mm, ovate-oblong. Lower lip: three united petals, 0.75–2.5 mm, obcordate, spreading; two lateral petals very short and entire or absent, the median petal up to 2–3 (−4) mm, bifid or undulate margin. Stamens: four—two anterior (4–5 mm long) and two posteriors (1–2 mm long), anthers 0.75–1 × 0.5 mm, dorsal, pubescent, and belly arched. Fruit: four nutlets, 1.5–3.0 × 1.0–1.5 mm, ovate-trigonous, smooth, acute base, pericarp brown with dense-sparse whitish spots. Flowering occurs in Egypt from December to February.

### 2.2. Inflorescence Heteroblasticity in the Egyptian Environment

**Figure 1:**
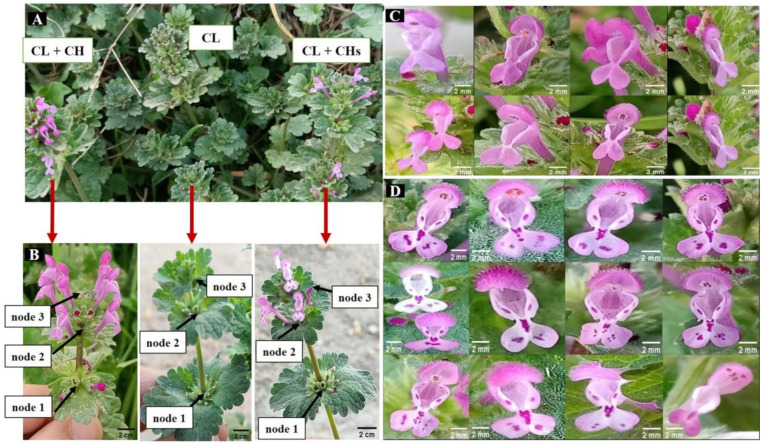
General features of the traced *L. amplexicaule* morphs (**A**) Three morphs coexist in the same population in the winter season (**B**) Virtual plasticity in the proportions of CL and CH flowers/node/morph (**C**) Corolla of the CH flowers in CL + CH morph showed diversity in color, size, and shape (**D**) Corolla of the CH flowers in CL + CHs morph showed diversity in color, size, shape and the number of spots.

The study on *L. amplexicaule* populations (80 individuals/morph from 35 populations) revealed the Presence and coexistence of three distinct morphs in the mixed population (Figure 1A) and at the same time during the winter season. The flowers were investigated in 8 nodes/branches, and the nodes were arranged in acropetal succession. The CL individuals (true cleistogamy), which carried only CL flowers and produced seeds in an autogamous manner; CL + CH (i.e., dimorphic cleistogamy); and other individuals that had both CL + CH flowers with spotted lower lips (dimorphic cleistogamy with spots; CL + CHs). This study revealed the predominance of the CL + CH morph when compared to the CL morph in Egypt. And the field observations in nearly all the studied populations showed that the (CL + CH, CL +CHs, and CL) morphs constituted 50%, 30%, and 20% of the mixed populations, respectively. These morphs showed plasticity in the percentages of both the CL and CH flowers/nodes/morphs (Figure 1B). The CH flowers of the Cl + CH and CL + CHs morphs are outlined in (Figure 1C,D), respectively, while the entirely CH individuals were not detected.

**Figure 2:**
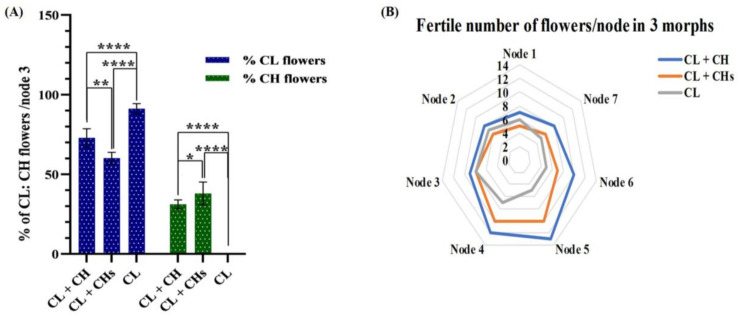
Diversity in CL: CH flowers in different nodes/morphs and its statistical model (**A**) Percentage of CL: CH flowers in node 3/morph. *p*-value: * = 0.021, ** = 0.002 and **** ≤ 0.0001 (**B**) Statistical model showing the number of total fertile flowers (CL and CH)/node/morph.

At node 3, the variation in the percentages of CL and CH flowers/node/morph, the percentage of CL flowers was 91.3%, 73.8%, and 60.2% in the CL, CL + CH, and CL + CHs morphs, respectively (Figure 2A), reflecting significant variations. Generally, the total number of flowers was the highest and lowest in the CL + CH and CL morphs, respectively. The highest number of flowers/nodes among the studied morphs were on node 5 (13 flowers) and node 4 (12 flowers) of the CL + CH morph. Conversely, the CL + CHs morph had a lower number of fertile flowers among all the morphs on nodes 1 and 2 (5 and 6 flowers, respectively; Figure 2B).

### 2.3. Lamium Phenoplasticity among the Morphs Using Macromorphological Characteristics

A morphological study was conducted on populations for the three morphs (Cl, CL + CH, and CL + CHs) using 131 macromorphological characteristics. There was substantial morphological plasticity among the traced morphs as follows:

- Cleistogamous morph (only CL flowers/individual); is characterized by ovate, non-segmented lowers leaves, short petiole (to 20 mm), and a larger inflorescence bract (27 mm × 30 mm) with shallow incised margin, and up to seven nodes with flowers/branches.
- Dimorphic-cleistogamous morph (CL + CH flowers/individual); its lower leaves are ovate-peltate, non-segmented, and have a longer petiole (to 27 mm). The inflorescence bract is similar to the CL morph in size, with a shallow-deeply incised margin and up to eight nodes with flowers/branches. The shape of the lower lip and the features of the lower corolla’s lateral lobes also showed variability (Figure 1C).
- Dimorphic-cleistogamous spotted morph (both CL + CHs flowers/individual with spotted petals) is characterized by a wide range of pink spotted lower lips (Figure 1D), and its lower leaves are ovate 3–5 segments. Petiole up to 30 mm long, the inflorescence bract is smaller than in the other morphs (its length to 20 mm) with shallow incised margin and up to ten nodes with flowers/branches.

### 2.4. Flower Phenoplasticity in the Different Morphs

#### 2.4.1. Calyx

**Figure 3:**
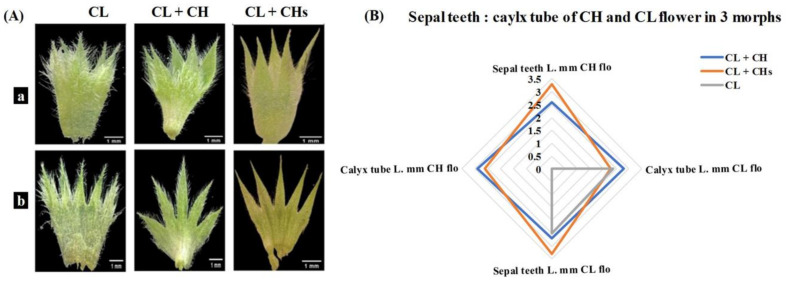
Calyx in the three morphs (**A**) Calyx feature where a: whole sepals and b: dissected sepals (**B**) Statistical model showing the sepal teeth: calyx tube in the three morphs.

(Figure 3A) shows the calyx in the CL morph sepals covered with dense trichomes all over, its triangular teeth cut to less than ½ tube length. While in the CL + CH morph, the trichomes are denser on the tube, and its lanceolate teeth cut to ½ tube length. Finally, the trichomes on the calyx of the CL + CHs morph are sparse on the teeth- and tube-main veins, and teeth narrow triangular cut to ½ tube length. The radar plot (Figure 3B) shows the length of the sepal teeth in the CH flower in the CL + CHs (3.3 mm) morph is larger than the corresponding teeth in the CL + CH (2.6 mm). While the length of the calyx tube is 2.9 mm and 2.6 mm; for the CL + CH and CL + CHs, respectively.

#### 2.4.2. Corolla

**Figure 4:**
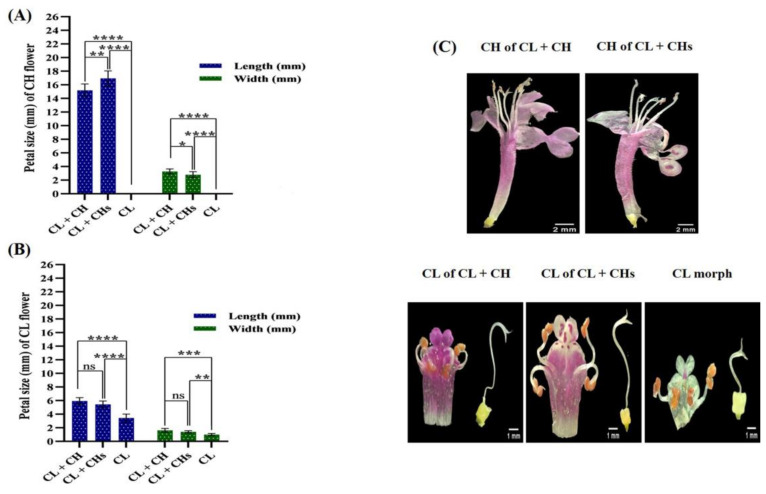
Petals features in the three morphs (**A**,**B**) Petal size of the different flowers (**C**) Heterostyly in the CL and CH flowers/morph. *p* value: * ≤ 0.05, ** ≤ 0.01, *** ≤ 0.001 and **** ≤ 0.0001. ns: not significant.

The petal length of the CH flowers in the CL + CHs (17.12 mm) is more significant than that in the CL + CH morph (14.88 mm), while the latter possessed wider petals (3.27 mm) when compared with those of the CL + CHs morph (2.71 mm). The petal size (length × width) of the CL flowers showed non-significant differences between the CL + CH and CL + CHs morphs (5.93 × 1.62 and 5.63 × 1.31 mm, respectively). (Figure 4A,B) outlined the diversity in petal length of the CH and CL flowers; the lengths of the petals in the CH flowers of the CL + CHs were significantly longer. While the lengths of the petals in the CL flowers of the CL morph (3.20 mm) were significantly shorter when compared with the CL flowers in the other morphs (5.9 and 5.6 mm, respectively). Four didynamous stamens (heterostyly) were observed in the studied three *L. amplexicaule* morphs (Figure 4C).

#### 2.4.3. Anthers

**Figure 5:**
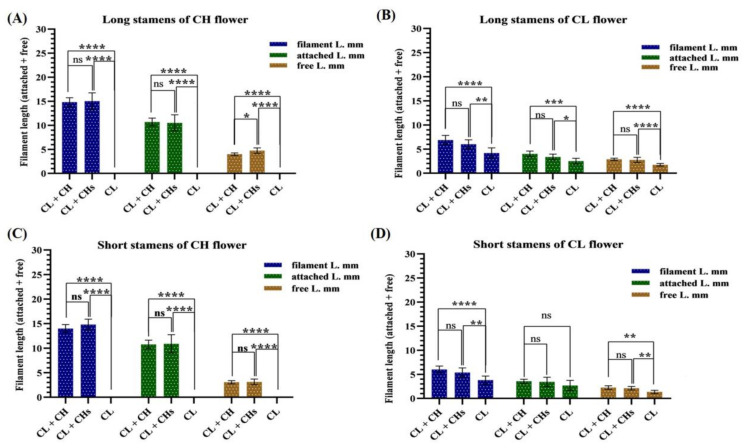
Stamens features in the three morphs (**A**–**D**) Diversity in stamens filament (short and long). *p* value: * ≤ 0.05, ** ≤ 0.01, *** ≤ 0.001 and **** ≤ 0.0001. ns: not significant.

(Figure 5A–D) outlined the diversity in filament (long & short) length in different flowers in the three morphs. There was no significant difference between the whole filament length (attached and free parts) between the studied morphs (Figure 4A). There were significant differences in the filament length of both the long (Figure 4B) and short stamens (Figure 4C) in the CL flowers of the CL morph (3.91 mm) compared to the equivalent flower in other morphs.

#### 2.4.4. Nectary Disk

The nectary disk of the CH flowers in the CL + CH morph (0.2–0.5 mm) was significantly longer than in the other morphs, while it showed a pronounced reduction in the CL flowers (0.1 mm).

#### 2.4.5. Pollen Fertility

**Figure 6:**
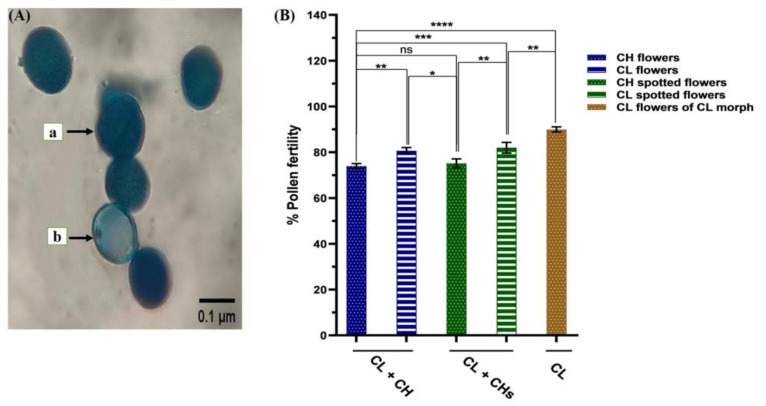
Pollen fertility in the three morphs (**A**) Pollen grains using Lactophenol cotton blue stain (a: Fertile pollen & b: Sterile pollen) (**B**) Percentages of pollen grain fertility in CL and CH flowers/morph. *p* values: * = 0.014, ** = 0.003, *** = 0.0003 and **** ≤ 0.0001 and ns: non-significant effect at >0.05.

Pollen productivity was highest in the CL + CH morph and lowest in the Cl morph. While the detected pollen fertility (Figure 6A) in the studied *Lamium* morphs ranged from 75–90% (count on microscope stage). Pollen fertility criterion varied significantly between the CH flowers and Cl flowers. It was nearly similar (c. 74%) in CH flowers and possessed similar pollen fertility in both morphs, while it was the highest (90.12%) in the CL flowers of the CL morph (Figure 6B).

#### 2.4.6. Nutlets Sculpture

**Figure 7:**
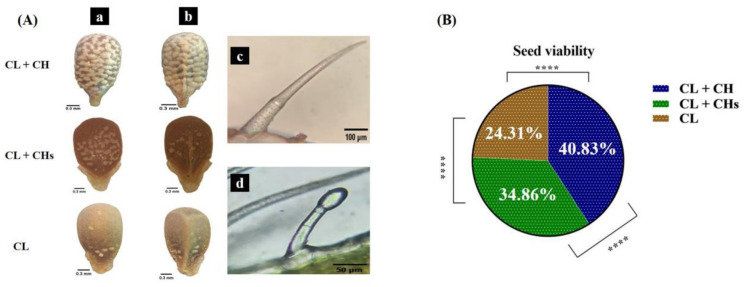
Nutlets, trichomes, and seed viability in the three morphs (**A**) Nutlets (a: dorsal view & b: ventral view) and trichomes (c: non-glandular & d: glandular) (**B**) Seed viability. ****: *p*-value ≤ 0.0001.

The *L. amplexicaule* fruit was split into 4 ovate-trigonous nutlets/flowers, each small size (1.5–3.0 × 1.0–1.5 mm) and covered with a dry-leathery non-spilling spotted pericarp (Figure 7A).

### 2.5. Trichomes

The indumentum examination using a light microscope showed the presence of non-glandular trichomes in the three morphs, covering all the organs with different densities (for example, sepals as shown in Figure 3). These trichomes were multicellular (3-cells) and ranges in length from 450–500 µm in all morphs Figure 7(Ac). For sepals, the non-glandular trichomes were mixed with the multicellular glandular capitate trichomes, the length of the latter ranges from 130–150 µm in all morphs. The length of the multicellular stalk (3 cells) is triple as long as the unicellular capitulum Figure 7(Ad).

### 2.6. Seed Viability and Morph Resemblance to Its Parent Seeds

The percentage of seed viability deduced from the 100 germinated seeds showed significant differences between the studied morphs (Figure 7B). The cultivated seeds/morph produced the first offspring/morph congruent with the morph of its parent seeds in percentages of 100%, 100%, and 98% for the CL + CH, CL + CHs, and CL morphs, respectively.

### 2.7. Variability in Flowering Timing among Morphs

Variability in flowering timing was observed between the studied morphs. However, all seeds were cultivated in October, and the CL morph was flowering earlier in mid-December. The other morphs entered the flowering stage in early- and mid-January for the CL + CHs and CL morphs, respectively. At the end of January, while the CL morph was fruiting, 2% of the individuals produced spotted chasmogamous flowers (CHs), and approximately 2.0–2.3% of the flowers/individual were in the second terminal node.

### 2.8. Genetic Diversity between the Studied Morphs

**Figure 8:**
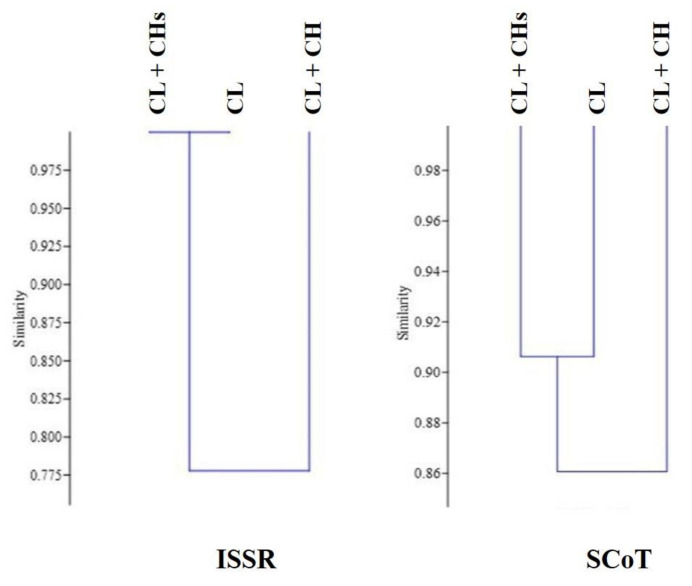
UPGMA dendrograms based on ISSR and SCoT markers.

The six ISSR primers produced 47 bands across the three morphs, of which 5 were polymorphic and three were unique bands. All the unique bands were exclusive to the CL + CH morph. The fragment lengths were 500 bp, 510 bp, and 600 bp for the primers 49A, 89B, and HB-11, respectively. Three primers did not show any variation between the studied morphs. The genetic similarity was calculated by Jaccard’s coefficient-SPSS program (Table 1). It showed that the genetic similarity of the CL + CHs morph was the highest with the CL morphs (100%), while it decreased to 0.77% with the CL + CH morph (Table 1). The genetic relationships between the morphs are shown in (Figure 8).

The seven SCoT primers produced 90 bands across the three morphs, of which 9 were polymorphic, and three produced unique bands. The CL morph characterized two unique bands at 1630 bp and 1345 bp for the primers SCoT 2 and 3, respectively. In comparison, one fragment with a length of 165 bp (SCoT 8) was unique to the CL + CH morph. Three primers did not show any variation between the studied morphs. The genetic similarity calculated by Jaccard’s coefficient-SPSS program showed that the genetic similarity of the CL morph was highest with the CL + CHs morphs (0.906%). In comparison, it decreased to 0.818% with the CL + CH morph, and the CL + CH morph was the base of the two other morphs (Figure 8 and Table 1).

## 3. Discussion

### 3.1. Inflorescence Heteroblasticity in the Egyptian Environment

This is a pioneer study including extensive revision of the heteroblastic inflorescence in *L. amplexicaule* L. It revealed the presence of two flower types representing two mating systems, chasmogamy (CH opened) and cleistogamy (CL closed), which has been previously reported for this species [4,10,14,23,24]. These two mating systems coexist in the same population in the same winter season under the Egyptian environment; a similar observation was reported in Japan, where *L. amplexicaule* exhibited two types of flowers (CL and CH) that coexisted on the same individual plant [11]. Based on the morphometric characters the studied species grouped into three morphs. These morphs were as follows: (1) the cleistogamous morph, which carried only CL flowers on an individual plant; (2) the dimorphic-cleistogamous morph, which carried both CL and CH flowers on an individual plant; and (3) the dimorphic-cleistogamous with spots, which carried both CL and CH flowers on an individual plant but exhibited additional dark violet spots on the corolla of the CH flower (in various forms and densities. The production of the CL and CH flowers in the same individual simultaneously is termed “pseudo-cleistogamous” [11]. Later, this term was renamed “dimorphic cleistogamy” [25], replacing previously used terms, such as pseudo-, true, and facultative cleistogamy.

### 3.2. The Growing Season of L. amplexicaule

The current revision detected the growth of *Lamium* in October (autumn) and identified its prevalence as a winter weed in farmland, which was in line with [26]. Analogous timing was reported in Southern USA, contrary to seasonality, as annual summer plant was reported in western Canada, in addition to its growth in spring and Autumn in Kentucky USA [9].

### 3.3. Predominant Morph in Egypt

This study revealed the predominance of the CL + CH and CL + CHs morphs over the CL morph in the winter season. However, opposite observations for the same species were reported in California-USA and northern Europe during the winter [10], where the CL flowering plants were predominant, regardless of the plant age, and the CH flowers emerged beside the CL flowers at the beginning of March. The production of the CL flowers was induced by the cool short days of winter while the long warm days in spring and summer enhanced the production of the CH flowers” [23].

### 3.4. Proportions of the CH: CL Flowers in Different Morphs

This study has pioneered dealing with the proportions of the CH and CL flowers at the infra-specific level (cleistogamous and dimorphic-cleistogamous morphs). The percentage of CL and CH flowers showed remarkable plasticity among the morphs. The highest number of flowers was in the CL + CH morph, while the CL morph was the lowest. The highest recorded number of flowers/nodes was 13 for node 5 of the CL + CH morph; a similar number was reported earlier [10].

The coexistence of the cleistogamous (CL) morph with the two dimorphic-cleistogamous morphs (CL + CH and CL + CHs) is congruent with the observations for the same species during the spring period in northern California, where individuals with up to 50% CH flowers were growing next to those bearing only CL flowers [10]. No available literature about the percentages of CL and CH flowers for this species; accordingly, the authors have no explanations to clarify the Presence of CL individuals within the mixed (CL + CH) individuals in one population. The studied *Lamium* morphs (CL, CL + CH, and CL + CHs) were comparable in size (ranging from 30–63 cm). Non-consistent data have been reported in other species, where the CH-production was linked to the plant size in *Viola soraria* [27], *Danthonia spicata* [28], and *Mimulus nasutuls* [29]. As reported earlier, the CH flower size was similar in all the studied morphs, regardless of the node position from which they arose [10].

### 3.5. Factors Controlling the Proportions of the CH Flowers

The current study also showed the presence of concurrent different proportions of the CH and CL flowers in different morphs. However, the researchers attributed this to the differences in day length and temperature [30], nutrient availability [31], and light aspects [23,25,31]. The concurrent difference in the percentage of CH flowers/individual in the two morphs (Cl + CH and CL + CHs), was attributed by earlier researchers to the seasonal pollinator activities and environmental conditions [14].

### 3.6. Flower Phenoplasticity in the Different Morphs

The current investigation revealed that the size of the CL flowers in the studied morphs gradually increased in the acropetal succession (data not shown), which was consistent with the other findings [10]. This study showed notable differences between the nectary disk, where the CL flowers host smaller nectary disks than the CH flower. These findings are consistent with those of previous studies [6,11,25]. The CL flower has previously been reported to have a reduced corolla and stamen size compared to the CH flower [25].

### 3.7. Pollen Grain Fertility

The pollen grain investigation indicated that the CL flowers in all the studied morphs possessed the lowest number (mean value of the pollen grain/anther 13/microscope stage); the CL morph showed the highest pollen fertility. While the CH flowers possess the lower fertility (mean value of the pollen grain/anther 24/microscope stage). The CL anther contains fewer pollen grains derived from a reduced number of pollen grain mother cells [32].

### 3.8. The Nutlet Sculpture and Seed Viability

The nutlet sculptures of the studied three morphs showed high levels of variability, which was consistent with [26]. The CL + CH individuals produced dense-spotted nutlets with the highest seed viability levels. While the CL morph produced unspotted nutlets with lower seed viability. 15% of the population had unspotted seeds [26], which was nearly consistent with this study’s percentage for the CL individuals/populations (20%). However, this study revealed that the CL + CH individuals had higher levels of seed viability while the CL individuals had the lowest levels. Seed viability patterns may differ in other cleistogamous species.

### 3.9. Trichomes

The epidermal examination of the studied morphs revealed the presence of multicellular non-glandular trichomes covering all the organs of the three morphs in different densities. Its length was very close to that cited for this species [1]. The previous studies reported the presence of non-glandular trichomes only in this species, among them [33]. Only, [1] reported the presence of the multicellular glandular trichomes; this is congruent with the results outlined in Figure 7(Ad). Unfortunately, not enough available data about the trichome micromorphology at the morphs level for comparison.

### 3.10. Morph Resemblance to Its Parent Seeds

The results revealed that the first offspring in the studied *L. amplexicaule* morphs were congruent to that of the parent-flower type (CL + CH, CL + CHs, or CL). Similar observations were reported for this species [14] and *Portulaca oleracea* [34], which suggested that this dimorphism in flower production may have a genetic basis.

### 3.11. Flowering Time for the Studied Morphs

The field observations during this investigation found that the flowering time for the CL morph was ten days earlier than for the dimorphic-cleistogamous morphs (CL + CH and CL + CHs), which meant that they flowered in early January (CL + CH) and mid-January (CL + CHs). Earlier studies supported these findings and reported that CL flowers were fruited earlier than CH [25,32]. The CL flowers may thus have advantages over the CH flowers when there are unfavorable environmental conditions [34]. This does not apply to all dimorphic-cleistogamous species; however, as with *Viola pubescens,* the CH flowers are produced earlier than the CL flowers [25].

### 3.12. Genetic Diversity with and within the Studied Morphs

However, previous studies have investigated the genetic diversity of the *Lamium* species [4,14,24,35,36], and the genetic distance between the cleistogamous and the dimorphic-cleistogamous of *L. amplexicaule* individuals has not been identified. The genetic distance between the CL and CH populations was studied in other species as *Viola pubescence,* using ISSR markers [37].

The genetic distance retrieved from the ISSR markers showed the highest similarity (1.0%) between the CL + CHs and CL morphs. This result may be related to our field observation at the end of January, where individuals of the fruiting CL produced spotted chasmogamous flowers (2% of CHs flowers; 2.0–2.3% flowers/individual). Seventy-three accessions from eight *Lamium* taxa were evaluated using ISSR markers, and these data were congruent with the observed morphological data [24]. The SCoT primers showed differential features and three unique bands, which were found to characterize the CL morph differently from the others. This pioneering study used SCoT markers to assess the *Lamium* species; the results indicated that SCoT markers might be able to efficiently delimit the *L. amplexicaule* morphs.

The deduced genetic distances were consistent with the retrieved morphological differences. The larger the genetic variations in a weed population, the better its chances of adapting to changing environments [35].

## 4. Materials and Methods

### 4.1. Plant Material

This study was based on an examination of *Lamium amplexicaule* (that possesses heteroblastic inflorescence) specimens kept in the Egyptian herbaria, Cairo University (CAI), Agricultural Museum (CAIM), National Research Centre (CAIRC), and Desert Research Institute (CAIH) to trace the species’ geographic distribution in Egypt. As well as to determine the different morphs of the mating systems, namely cleistogamous (CL), and dimorphic-cleistogamous (CL + CH) individuals in relation to their collection time. Voucher specimens for the studied morphs were deposited in the CAI and Beni-Suef Herbarium. The locations of the studied specimens/populations (herbarium and fresh) are outlined in (Table 2). Field studies were conducted at five localities designated with stars in (Table 2) during the winter season of two successive years (2021 and 2022). A total of 80 individuals/morphs from 35 populations (8 populations/locality) were kept in the Cairo University Herbarium (CAI) laboratory for future investigation. Fresh samples were preserved in formol-acetic-alcohol (FAA: 50 mL ethyl alcohol, 10 mL formaldehyde, 5 mL glacial acetic acid, and 35 mL distilled water) for further investigations.

#### 4.1.1. Morphological Investigations, Seed Viability, and Morph Resemblance to the Parental Seeds

The collected materials (a total of 80 individuals/morph from 35 populations and 10 flowers/individual) were morphologically investigated using 131 macromorphological characteristics (including stem, leaf, inflorescence, flowers, fruit, seed, and pollen fertility). While seed viability of the detected flower morphs (CL, CL + CH, and CL+ CHs), 100 full-ripened seeds were collected from each morph (80 individuals/morph) according to [38] in February 2021 and germinated (five replicates/morph) in an open field of the Beni-Suef experimental garden in December 2022. To maintain viability, irrigation was provided when required [39]. The seedling percentages from each morph were calculated and left to achieve full flowering to monitor and detect the offspring-morph consistency with its parental seed morph. The nutlet sculpture can also provide potential data to help delimitate the *Lamium* species and hybrids [40].

#### 4.1.2. Pollen Grain Fertility

The pollen fertility test was carried out using Lactophenol cotton blue to stain the pollen from the different detected morphs (ten anthers from ten flowers harvested from the upper first three nodes/morph), according to [41]. Lactophenol cotton blue is an efficient tool to separate male-sterile hybrids from fertile pollen [42]. A light microscope (AmScope M150C-I 40X-1000X) was then used to study pollen fertility; the fertile pollen was stained dark blue, while the sterile (aborted) pollen appeared pale blue, and the percentage of pollen fertility was determined as described by [43].

#### 4.1.3. Molecular Investigation

Genomic DNA was isolated from the freshly harvested juvenile leaves using a DNeasy plant mini kit (bio basic) and used as a template for polymerase chain reaction amplification using six ISSR primers and seven SCoT primers (the positive primers were selected from 15 pretested markers), the sequences are outlined in (Table 3).

Polymerase Chain Reaction (PCR): Genomic DNA was used as a template for PCR amplification using 5 ISSR primers and 6 SCoT primers in molecular assessment for the juvenile leaves specimens. ISSR primers procured from Operon Technology, Alameda, U.S.A. On the other hand, SCoT primers were designed from a consensus sequence derived from earlier studies [19,44]. All SCoT primers were 18-mer and were from Dataset I, which is based on highly expressed genes, according to [45]. For SCoT primers design, the start codon ATG (+1, +2, and +3), ‘G’ at position +4, ‘C’ at position +5, and A, C, C, and A at positions +7, +8, +9, and +10, respectively, were fixed (5′ ---ATGGCTACCA---3′). The ISSR and SCoT amplification reaction techniques were performed as described by [46,47]. Amplification reactions were carried out in Techni TC-512 Thermal Cycler as follows: One cycle at 94 °C for 4 min followed by 40 cycles of 1 min at 94 °C, 1 min at an annealing temperature of 57 °C for 2 min at 72 °C, followed by 72 °C for 10 min, the reaction was finally stored at 4 °C. DNA banding pattern photos were photographed using the Bio-1D Gel Documentation system. They were analyzed by Gel Analyzer 3 software of version 19.1, which scored clear amplicons as present (1) or absent (0) for the ISSR and SCoT primers, according to [48].

### 4.2. Statistical Analysis

The field data were tested for normality using the Kolmogorov-Smirnov test. This test showed that all data exhibited normality. The resulting morphological data, recorded as (means values), were analyzed and plotted using GraphPad Prism software version 8.4.2. One-way analysis of variance (ANOVA) was used for all statistical comparisons, and Tukey’s post hoc test analysis was performed (*p* < 0.05 was considered significant, while *p* > 0.05 was considered non-significant).

Genetic similarity between the studied morphs was calculated using Jaccard’s coefficient-SPSS program (version 20 for Windows). A dendrogram was generated by cluster analysis using the unweighted pair group method of the arithmetic averages (UPGMA) using Past software (version 3.26 for Windows). Similar output was also retrieved using the Genetic Similarity Coefficient using the Dice Coefficient formula (GSij = 2a/(2a + b + c).

## 5. Conclusions

This pioneering study covered the heteroblastic inflorescence of *L. amplexicaule* L. representing the genus *Lamium* L. in Egypt. This species was traced in three morphs (CL, CL + CH, and CL + CHs). The relation between these morphs was clarified with ISSR and SCoT molecular markers. This study provides information about the flower features in the different morphs, flowering time, pollen productivity and fertility, nutlets sculpture, productivity, and viability, in addition to the genetic fingerprint of the different morphs. All these data may help to eradicate this cosmopolitan and invasive crop weed and provide a template for the species with similar heteroblastic inflorescence.

## Figures and Tables

**Table 1:** Genetic similarity between the studied morphs using Jaccard’s coefficient.

Using SCoT Results			
	CL + CH	CL + CHs	CL
**CL + CH**	1.000	0.903	0.818
**CL + CHs**	0.903	1.000	0.906
**CL**	0.818	0.906	1.000
**Using ISSR results**			
**CL + CH**	1.000	0.778	0.778
**CL + CHs**	0.778	1.000	1.000
**CL**	0.778	1.000	1.000

**Table 2:** Localities for the studied *Lamium amplexicaule* specimens. (Localities arranged from north to south).

Locality	GPS Coordinates		Date of Collection
	N°	E°	
Metobus-Kafr el Shiek *	31°29′75″	30°52′33″	20 February 2021 & 9 March 2022
Barrage-Damietta	31°24′18″	31°47′17″	7 January 1927
Barrage-Damietta	31°23′18″	31°47′17″	2 April 1950 & 30 March 1956
Behiera Province	31°24′03″	30°25′00″	25 April 1987
Samouha-Alexandria	31°12′56″	29°56′30″	23 March 1956
Dikirnis-Dakahlia	31°06′52″	31°38′55″	21 May 1967
Mansoura	31°02′29″	31°22′41″	19 March 1974
Tanta	30°47′11″	31°00′01″	12 March 1968
El-Salhiya, Sharkiya	30°44′51″	32°00′18″	1 October 1983
Shubrakhit-Behiera	30°41′17″	30°14′28″	5 March 1988
Ismailia	30°35′17″	32°16′26″	18 March 1927
Mit Kinana-Qaliubia	30°23′08″	31°15′42″	14 February 1969
Abu-Zaabal-Cairo	30°14′27″	31°21′10″	9 April 1954
Mattaria-Cairo	30°07′35″	31°19′02″	February 1952
Mattaria-Cairo	30°07′34″	31°19′03″	1 May 1949
Abu Sleem El-Menoufia *	30°06′45″	31°12′54″	30 January 2022 & 27 January 2022
Zaafran palace-Cairo	30°04′31″	31°17′03″	24 March 1926
Orman Garden-Cairo	30°01′45″	31°12′46″	5 April 1928
Cairo University-Giza	30°01′39″	31°12′27″	7 January 1952
Cairo University Garden-Giza	30°01′37″	31°12′33″	1971
Faculty Agriculture-Giza	30°01′09″	31°12′38″	5 April 1971
Giza spring garden-Giza	30°00′53″	31°12′32″	1962
El Saf-Giza *	29°34′57″	31°15′17″	15 November 2021 & 10 January 2022
El-Siliene spring-Faiyum *	29°24′48″	30°51′27″	2 December 2021 & 18 January 2022
Faiyum fields-Faiyum	29°18′27″	30°36′29″	1952
Qoshesha-Benisuef	29°17′53″	31°10′37″	19 March 1982
Belyfa-Beniseuf *	29°07′27″	31°02′56″	20 January 2021 & 18 February 2022
El-sheik Awad-S. Sinai	28°52′01″	33°00′14″	20 February 2018
Deir el Arba’-S. Sinai	28°47′25″	33°35′23″	12 May 1956
Deir el Rabba-S. Sinai S	28°33′25″	33°58′23″	25 April 1961 & 24 May 1961
Saint Catherine Monastery-S. Sinai	28°33′25″	33°58′23″	11 April 1967
Tell El Amarna-Minya	27°38′41″	30°53′55″	22 January 1968
Hurghada-Red Sea	27°13′36″	33°46′07″	10 February 1961
Dakhla Oasis-Western desert	25°32′30″	28°55′47″	11 February 1931
Kharga Oasis-Western desert	25°26′30″	30°33′47″	13 February 1952

*: collected samples.

**Table 3:** Data were retrieved from the ISSR and SCoT primers.

Primer Code	Primer Sequence	No. of Total Amplified Bands			No. of Polymorphic Bands			No. of Unique Bands			Polymorphism%/Primer		
		CL + CH	CL + CHs	CL	CL + CH	CL + CHs	CL	CL + CH	CL + CHs	CL	CL + CH	CL + CHs	CL
**SCoT primers**													
**SCoT 2**	ACC ATG GCT ACC ACC GGC	2	3	5	0	1	3	0	0	2	0	33.33	60
**SCoT 3**	ACG ACA TGG CGA CCC ACA	6	6	6	0	0	0	0	0	0	0	0	0
**SCoT 4**	ACC ATG GCT ACC ACC GCA	4	4	3	1	1	0	0	0	0	25	25	0
**SCoT 6**	CAA TGG CTA CCA CTA CAG	1	2	2	0	1	1	0	0	0	0	50	50
**SCoT 8**	ACA ATG GCT ACC ACT GAG	3	2	2	1	0	0	1	0	0	33.33	0	0
**SCoT 10**	ACA ATG GCT ACC ACC AGC	5	5	5	0	0	0	0	0	0	0	0	0
**SCoT 14**	ACC ATG GCT ACC AGC GCG	8	8	8	0	0	0	0	0	0	0	0	0
**ISSR primers**													
**49 A**	5′ CAC ACA CAC ACA AG 3′	3	3	3	1	1	1	1	0	0	33.33	33.33	33.33
**89 A**	5′ CAC ACA CAC ACA CA 3′	3	3	3	0	0	0	0	0	0	0	0	0
**89 B**	5′ CAC ACA CAC ACA GT 3′	4	3	3	1	0	0	1	0	0	25	0	0
**HB-10**	5′ GAG AGA GAG AGA CC 3′	2	2	2	0	0	0	0	0	0	0	0	0
**HB-11**	5′ GTG TGT GTG TGT TGT CC 3′	2	1	1	1	0	0	1	0	0	50	0	0
**HB-12**	5′ CAC CAC CAC GC 3′	3	3	3	0	0	0	0	0	0	0	0	0

## Data Availability

All data available through the manuscript.

## Abbreviations

CL + CH: dimorphic cleistogamy; CL + CHs: dimorphic cleistogamy with spots; CL: true cleistogamy; CH flower: chasmogamous flower; CHs: chasmogamous with spots flower; CL flower: cleistogamous flower and flo: flower.
